# Fucoidan-Stabilized Gold Nanoparticle-Mediated Biofilm Inhibition, Attenuation of Virulence and Motility Properties in *Pseudomonas aeruginosa* PAO1

**DOI:** 10.3390/md17040208

**Published:** 2019-04-03

**Authors:** Fazlurrahman Khan, Panchanathan Manivasagan, Jang-Won Lee, Dung Thuy Nguyen Pham, Junghwan Oh, Young-Mog Kim

**Affiliations:** 1 Marine-Integrated Bionics Research Center, Pukyong National University, Busan 48513, Korea; fkhan055@pknu.ac.kr (F.K.); manimaribtech@gmail.com (P.M.); jungoh@pknu.ac.kr (J.O.); 2 Department of Food Science and Technology, Pukyong National University, Busan 48513, Korea; ananias93@naver.com (J.-W.L.); dungpham0495@gmail.com (D.T.N.P.); 3 Department of Biomedical Engineering, Pukyong National University, Busan 48513, Korea

**Keywords:** antibiofilm, fucoidan, motility, nanoparticles, *Pseudomonas aeruginosa*, virulence factors

## Abstract

The emergence of antibiotic resistance in *Pseudomonas aeruginosa* due to biofilm formation has transformed this opportunistic pathogen into a life-threatening one. Biosynthesized nanoparticles are increasingly being recognized as an effective anti-biofilm strategy to counter *P. aeruginosa* biofilms. In the present study, gold nanoparticles (AuNPs) were biologically synthesized and stabilized using fucoidan, which is an active compound sourced from brown seaweed. Biosynthesized fucoidan-stabilized AuNPs (F-AuNPs) were subjected to characterization using UV-visible spectroscopy, Fourier transform infrared spectroscopy (FTIR), field emission transmission electron microscopy (FE-TEM), dynamic light scattering (DLS), and energy dispersive X-ray diffraction (EDX). The biosynthesized F-AuNPs were then evaluated for their inhibitory effects on *P. aeruginosa* bacterial growth, biofilm formation, virulence factor production, and bacterial motility. Overall, the activities of F-AuNPs towards *P. aeruginosa* were varied depending on their concentration. At minimum inhibitory concentration (MIC) (512 µg/mL) and at concentrations above MIC, F-AuNPs exerted antibacterial activity. In contrast, the sub-inhibitory concentration (sub-MIC) levels of F-AuNPs inhibited biofilm formation without affecting bacterial growth, and eradicated matured biofilm. The minimum biofilm inhibition concentration (MBIC) and minimum biofilm eradication concentration (MBEC) were identified as 128 µg/mL. Furthermore, sub-MICs of F-AuNPs also attenuated the production of several important virulence factors and impaired bacterial swarming, swimming, and twitching motilities. Findings from the present study provide important insights into the potential of F-AuNPs as an effective new drug for controlling *P. aeruginosa*-biofilm-related infections.

## 1. Introduction

The formation of biofilm by *Pseudomonas aeruginosa* contributes to its survival in adverse environmental conditions, defense against the host immune system, and resistance to antimicrobial compounds such as conventional antibiotics, resulting in extreme complications in preventing and eradicating this opportunistic pathogen from infected patients and medical facilities [1,2,3,4]. Apart from the formation of the biofilm matrix, several virulence factors are also produced, which further aid the bacteria in causing chronic infections [2,5]. With the rapid pace of emergence and spread of *P. aeruginosa* with biofilm-forming ability, current anti-biofilm and anti-virulence approaches have mainly targeted the following: (1) attachment of planktonic cells, (2) cell-to-cell communication networks and regulatory systems, and (3) eradication of pre-existing matured biofilm structures [6,7]. Furthermore, these modern anti-biofilm approaches highly favor treatments which are bioactive, cost-effective, and less toxic [8,9,10,11].

Recently, nanomaterials have become popular, owing to their various physiochemical advantages resulting from their nano-scale size, such as high surface area to volume ratio, low toxicity, and high stability [12,13]. The gold nanoparticle (AuNP) possesses these properties, and is one of the commonly-used nanoparticles, with several applications in catalysis, electronics, nonlinear optics, drug delivery, and disease diagnosis in medical fields [14,15,16,17,18]. In comparison with chemical methods, which employ surfactants in the synthesis of this nanoparticle (NP), biological methods employing ‘green’ materials such as biopolymers provide significant benefits in terms of reducing NP aggregation, production costs, simple isolation, and environmental friendliness [19,20,21,22]. The morphology regarding size, shape, and crystalline properties, as well as the biocompatibility and stability of biosynthesized AuNP, are also significantly improved [23]. Although several biological systems are currently used to synthesize NPs, edible marine algae are highly preferred due to their widespread availability and richness in bioactive compounds, which could act as active stabilizing and reducing agents [24]. The bioactive compound fucoidan used in the present study is a fucose-rich and sulfated polysaccharide present in diverse brown seaweed species. Fucoidan has been extensively utilized as an important antitumor, antibacterial, antiviral, anti-inflammatory, and antioxidant agent owing to its biodegradable, biocompatible, non-toxic, and water-soluble characteristics [25,26]. In efforts to overcome antibiotic resistance in bacteria, previous studies have shown that both biosynthesized AuNPs and fucoidan-synthesized-NPs exhibit high antibacterial activity towards a variety of bacteria [27,28,29]. Therefore, the present study aimed to synthesize and characterize fucoidan-stabilized gold nanoparticles (F-AuNPs), as well as to evaluate their application as a potential anti-biofilm and anti-virulence drug against *P. aeruginosa*.

## 2. Materials and Methods

### 2.1. Bacterial Strains, Culture Media, Chemicals, and Growth Conditions

The study was performed using *P. aeruginosa* PAO1 KCTC 1637 obtained from Korean Collection for Type Cultures, Daejeon, Korea as the reference strain. The liquid and solid media used for the growth and cultivation of *P. aeruginosa* were tryptic soya broth (TSB; Difco Laboratory Inc., Detroit, MI, USA) and tryptic soya agar (TSA) plate. The pH of the media was adjusted to 7.2. Fucoidan (≥95%) sourced from *Fucus vesiculosus*) and hydrogen tetrachloroaurate (III) were obtained from Sigma-Aldrich Co. (St. Louis, MO, USA). All the reagents and chemicals used in the present study were of analytical grade. The growth condition of *P. aeruginosa* was aerobic and the growth temperature was maintained at 35 °C throughout the experiment.

### 2.2. Synthesis and Characterization of F-AuNPs

The chemical synthesis and instrumental characterization of F-AuNPs were carried out according to the procedure described previously [30]. The F-AuNPs were synthesized by mixing fucoidan (5.0 mg) into a solution of HAuCl_4_.3H_2_O (1 × 10^−4^ M) at the temperature of 80 °C for 30 min under continuous stirring. The color change of the solution into dark ruby red was considered as an initial indicator of F-AuNP formation. Furthermore, F-AuNP formation was also monitored by measuring absorbance spectra using DU-530 spectrophotometer (Beckman Coulter, Fullerton, CA, USA). The resulting solution was centrifuged (12,000× *g* for 30 min), followed by washing with deionized water. The unreacted gold was dispersed into water and dialyzed using a 12,000 Da molecular weight cut-off dialysis tube for 24 h at room temperature in order to remove it from the mixture.

Different physiochemical properties, including size, morphology, stability and composition, of newly synthesized F-AuNPs were characterized using various instruments and methods. The morphology of F-AuNPs was determined using field emission transmission electron microscopy (FETEM) JEM-2100F (JEOL Ltd., Tokyo, Japan). The particle size of the F-AuNPs was measured using dynamic light scattering (DLS) with the help of an electrophoretic light scattering spectrophotometer (ELS-800, OTSUKA Electronic Co., Ltd., Osaka, Japan). The room temperature and fixed angle (90°) in the spectrophotometer were set for scattering and measuring the spectra. The elemental composition of F-AuNPs was determined using energy dispersive X-ray diffraction (EDX; Hitachi, S-2400, Tokyo, Japan). The functional groups of each component present in F-AuNPs were determined by Fourier transform infrared spectroscopy (FTIR). The FTIR of F-AuNPs was carried out in a diffuse reflectance mode with a range of wavelengths from 4000 to 400 cm^−1^. Finally, the crystalline structure of the F-AuNPs was examined using X-ray diffraction (XRD; X’Pert-MPD system, Philips, Almelo, The Netherlands).

### 2.3. Determination of Minimum Inhibitory Concentrations of F-AuNPs and Growth of P. aeruginosa Cells in the Presence of F-AuNPs

Minimum inhibitory concentration (MIC) was defined as the complete inhibition of bacterial growth with no visible turbidity by the action of F-AuNPs at the lowest concentration. Determination of MIC of F-AuNPs against *P. aeruginosa* PAO1 followed the guidelines from the Clinical and Laboratory Standards Institute (CLSI), 2016 [31]. Briefly, the cell culture of *P. aeruginosa* was grown overnight and then added to a 96 well microtiter plate. Two-fold serial diluted concentrations of F-AuNPs (1024 to 32 µg/mL) (10 mg/mL stock prepared in sterilized distilled water) were added to the plate. The plate was then incubated at 35 °C for 24 h under orbital agitation (120 rpm) in the microtiter plate reader (BioTek, Winooski, VT, USA). After incubation, the optical density (OD) of the grown bacterial cells at 600 nm was measured. Similarly, the growth property of *P. aeruginosa* in the presence of different concentrations of F-AuNPs was also measured using a similar method to that discussed above. The only difference was the measurement of OD of the grown cells, which was monitored at time intervals of every 2 h in the microplate reader. Both MIC and growth assays were performed in triplicate.

### 2.4. Crystal Violet Staining Method for the Biofilm Assays

The crystal violet method was used for the quantitative estimation of biofilm formation in the presence and absence of the compound, following the procedure described earlier [8]. The minimum concentration of F-AuNP that inhibited *P. aeruginosa* biofilm formation (minimum biofilm inhibition concentration: MBIC) was also determined. Briefly, the *P. aeruginosa* cell culture (grown overnight in TSB) was diluted to a turbidity of 0.05 at 600 nm, and then treated with different concentrations of F-AuNPs (ranging from 16 to 256 µg/mL). After 24 h of incubation at 35 °C, the planktonic cells were discarded, while the attached cells were washed three times with water and then stained with crystal violet (0.1%). After 20 min of incubation, the crystal violet dye was discarded and the attached cells were again washed thrice with water. The adhered cells were re-suspended with 95% ethyl alcohol followed by the OD determination at the wavelength of 570 nm. Simultaneously, the *P. aeruginosa* growth property in the presence of F-AuNPs was also determined in static conditions by measuring the OD at 600 nm. For both biofilm and growth analysis, each concentration of F-AuNPs was repeated three times.

Crystal violet assay was also performed to investigate the eradication effect of F-AuNPs on pre-formed matured *P. aeruginosa* biofilm. The minimum concentration at which F-AuNPs exhibited eradication effect on pre-formed matured biofilm (minimum biofilm eradication concentration: MBEC) was also determined. The first step was to allow the formation of biofilm for 24 h by incubating *P. aeruginosa* in TSB without F-AuNPs in the 96 well microtiter plate, as discussed earlier [8]. Briefly, after incubation, the planktonic cells were removed and attached biofilm cells were washed thrice with fresh TSB media. The established biofilm cells were treated with different concentrations of F-AuNPs (16–256 µg/mL) in fresh TSB media. The microtiter plate was then incubated at 35 °C for 24 h, and quantified for biofilm cells after staining with 0.1% crystal violet following the procedure described in detail in the biofilm assay section. The experiment was performed in triplicate.

### 2.5. Microscopic Examination of the Biofilm Formed Cells

Visualization of the cell morphology and biofilm architecture was carried out by using microscopes such as the scanning electron microscopy (SEM) and fluorescence microscopy. The procedure used for the SEM sample preparation was adopted as discussed earlier [8,32]. Briefly, the cell culture was allowed to grow in TSB media on the surface of nylon membranes (0.5 × 0.5 cm) placed in a 24 well microtiter plate in the presence and absence of F-AuNPs (256 µg/mL). The 24 well microtiter plate was incubated for 24 h at 35 °C. The biofilm cells were directly fixed by formaldehyde and glutaraldehyde and kept at 4 °C temperature overnight. After removing the unattached cells, the fixed cells were washed three times with phosphate buffer saline (PBS; pH 7.4), followed by dehydration in increasing concentrations of ethyl alcohol at 50, 70, 80, 90, 95 and 100%. The adhered cells on the nylon membrane were freeze-dried using a freeze dryer machine (FD8518, ilShinBiobase Co., Ltd., Dongducheon, Korea), followed by fixation to SEM stubs. The affixed membrane was further coated with gold for 120 s with the help of an ion-sputter (E-1010, Hitachi, Tokyo, Japan). The prepared samples were visualized for the study of cell morphology using JSM-6490LV (JEOL, Tokyo, Japan) at the magnification of ×5000 and voltage of 15 kV. Similarly, the biofilm architecture was also observed using a Leica DMI300B fluorescence microscope at a magnification value of ×40, as described earlier [8]. However, for the fluorescence microscope, (Leica Microsystems, Wetzlar, Germany), the sample was prepared on the glass pieces and was placed in a 6 well microtiter plate. Before visualization of the cells, the biofilm cells on the surface of glass pieces were washed three times with PBS, followed by staining with 10 µg/mL working concentration of acridine orange dye. The stained cells were again washed with PBS and observed under a fluorescence microscope.

### 2.6. Determination of Hemolytic and Protease Activities

The hemolytic property of *P. aeruginosa* in the presence of F-AuNPs was determined using the red blood cells (RBCs) following the procedure described previously [8,33]. Briefly, the *P. aeruginosa* cell culture was grown overnight and was then supplemented with different concentrations of F-AuNPs (ranging from 32 to 256 µg/mL) in a 96 well microtiter plate, followed by incubation at 35 °C for 12 h in shaking condition (120 rpm). The treated and non-treated bacterial cell cultures (50 µL) were mixed with diluted RBCs. A negative control was prepared by mixing the F-AuNPs (256 µg/mL) with diluted RBCs. The bacterial cell culture mixed with RBCs was incubated at 35 °C for 1 h in shaking incubator (120 rpm). The mixture was centrifuged at 16,600× *g* for 10 min, and the OD of supernatant containing hemolyzed RBCs was determined by measuring at 543 nm. The experiment was performed in triplicate.

The production and activity of the protease enzyme from *P. aeruginosa* were tested in the presence and absence of F-AuNPs on casein agar plate, as described in the previous protocol [8,33]. The casein agar plate was prepared by mixing casein powder (10%) into autoclaved Bacto agar (2%) in 100 mL distilled water. The filtered supernatant (10 µL), which was obtained from the overnight grown *P. aeruginosa* cell culture (initial turbidity of 0.05 at 600 nm) in the presence of different concentrations of F-AuNPs (16–256 µg/mL), was loaded in the holes of a casein agar plate. After 24 h of incubation at 35 °C temperature, diameters (cm) of the clear zones around the holes were measured to determine the inhibition of F-AuNPs to bacterial protease activity. Analysis of protease activity was performed in two replicates using two independent cultures.

### 2.7. Quantitative Estimation of Virulence Factor Production

The impact of F-AuNPs on the production of several virulence factors from *P. aeruginosa*, such as pyocyanin, pyoverdine, and rhamnolipid, was examined in the present study. The methodology of the assays of each virulence factor production was adopted from the previous protocol [8,33]. For the estimation of virulence factors production such as rhamnolipid and pyocyanin, TSB media was used, whereas for the estimation of siderophore-like pyoverdine, iron-limited minimal salt media (MSM) along with 2% sodium succinate (SS) was used. The cell culture (5 mL) of *P. aeruginosa* (initial turbidity of 0.05 at 600 nm) grown overnight was incubated with various concentrations of F-AuNPs in test tubes containing either TSB (for pyocyanin and rhamnolipid assays) or MSM + 2% SS (for pyoverdine assays), and was incubated under shaking condition at 35 °C for 12 h. After 12 h of incubation, for the pyocyanin estimation, the cell-free supernatant was mixed with chloroform for the extraction of green-blue colored pigment, as described in detail in a previous study [34]. The collected blue-green colored sample turned a pink color when it was acidified with HCl (0.2N), and was then quantified by measuring the OD at 520 nm. The rhamnolipid from the supernatant was extracted using an organic solvent i.e., diethyl ether, and the quantification was carried out by orcinol colorimetric method following the detailed procedure described earlier [35]. The total content of rhamnolipid was quantified by measuring the OD at 421 nm. For the estimation of pyoverdine, the supernatant was directly quantified by the OD at 405 nm, as discussed earlier [36]. All experiments were performed in triplicate.

### 2.8. Assays of Motility Properties of P. aeruginosa

The effect of F-AuNPs at sub-MICs on different types of motility such as swarming, swimming, and twitching of *P. aeruginosa* was tested as described previously [33,37]. Two sub-MIC levels were selected for all motility assays (32 µg/mL and 256 µg/mL). To check the swarming motility, the Bacto agar (0.4%) plate prepared in Luria Britani (LB) broth containing casamino acid (0.5%) and glucose (0.5%) was used. For swimming motility, the Bacto agar (0.3%) was also used, however, it was prepared in distilled water along with 1% NaCl and 0.25% tryptone. Each plate was also supplemented with different concentrations of F-AuNPs. The *P. aeruginosa* cell culture (10 µL) was grown overnight and then placed on the center of swarming and swimming agar plates, followed by incubation at 35 °C for 24 h. The experiment was repeated two times. The two movements were demonstrated by the zone of cell travelling on the agar after incubation for 24 h. The assay for twitching motility was slightly different compared to the swarming and swimming motilities, and was performed following the protocol described previously. For the twitching motility assay, the overnight grown cell culture (10 µL) was firstly stubbed a thin layer in the center of Petri dishes, followed by pouring of Bacto agar (1.5%) prepared in LB supplemented with glucose (30 mM) and casamino acid (0.2%). After 24 h of incubation, the total agar content was discarded and the cells attached to the surface of the plate were stained with crystal violet (0.1%), then were washed with water and air dried. The crystal violet stained area of the cells is the indicator of twitching motility. The assay of twitching motilities was also performed in replicates.

### 2.9. Statistical Analysis

All graphs in the present study were constructed using GraphPad Prism 7.0 (GraphPad Software Inc., San Diego, CA, USA). All data in the present study were obtained from one-way ANOVA and are represented as mean ± standard deviation.

## 3. Results

### 3.1. Synthesis and Characterization of F-AuNPs

**Figure 1:**
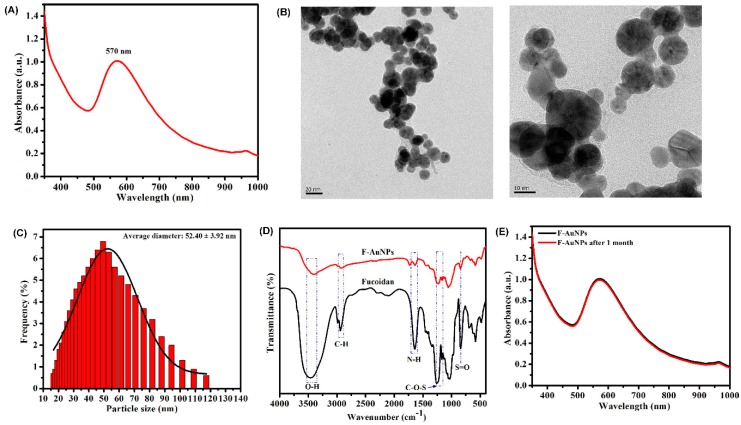
Synthesis and characterization of fucoidan-stabilized gold nanoparticles (F-AuNPs). (**A**) UV-visible-absorbance spectra of F-AuNPs, (**B**) field emission transmission electron microscopy (FE-TEM) image of F-AuNPs, (**C**) dynamic light scattering (DLS) histogram of particle size distribution, and (**D**) Fourier transform infrared spectroscopy (FTIR) spectrum of F-AuNPs, and (**E**) UV-visible absorbance spectra of the freshly synthesized and one-month old F-AuNPs.

F-AuNPs were synthesized by the reduction of ionic gold (Au^3+^) in a chloroauric acid solution with the help of fucoidan. Fucoidan, which is a negatively charged polymer derived mainly from marine seaweed, acts as a stabilizing and reducing agent. The initial indication and confirmation of F-AuNP synthesis were established by checking the appearance of ruby red color, as well as by measuring absorbance spectra using UV-visible spectrophotometry (Figure 1A). The maximum absorbance peak was found at 570 nm, which was almost coincident with the peak obtained (566 nm) during the synthesis of AuNPs by Manivasagan et al. [30].

The morphology of the synthesized F-AuNPs was characterized using field emission transmission electron microscopy (FE-TEM) (Figure 1B). The distribution of F-AuNP sizes was also determined using dynamic light scattering (DLS) (Figure 1C). The results of FE-TEM and DLS showed that F-AuNPs were spherical in shape and ranged in size from 15 to 119 nm; the average size of the particles was ~53 nm (Figure 1C). Furthermore, chemical interactions between different functional groups present in the polymeric fucoidan and AuNPs were determined by Fourier transform infrared spectroscopy (FTIR). The FTIR results (Figure 1D) demonstrated that fucoidan showed characteristic peaks at 845 cm^−1^ and 1159–1260 cm^−1^, corresponding to the S=O asymmetric stretching and C–O–S stretching of sulfate groups, respectively. The bands in the spectra at 1633 cm^−1^ and 1637 cm^−1^ in both fucoidan and F-AuNPs correspond to the N–H bending of amines. Similarly, the bands at 3441 cm^−1^ and 3444 cm^−1^ in both fucoidan and F-AuNPs spectra correspond to the O–H stretching of alcohol, whereas the bands at 2932 cm^−1^ and 2933 cm^−1^ spectra correspond to the C–H stretching of alkanes. Figure 1E represents the UV-visible absorbance spectra of freshly prepared and one-month old F-AuNPs.

**Figure 2:**
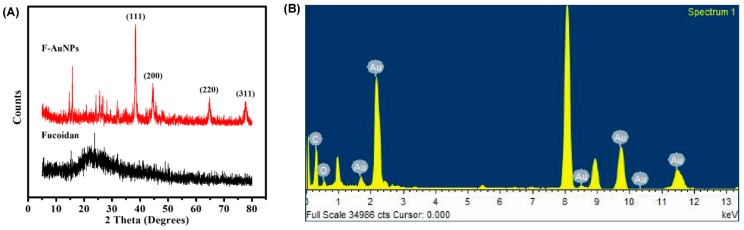
(**A**) The X-ray diffraction (XRD) pattern of F-AuNPs and (**B**) X-ray spectrum of the F-AuNPs.

Different diffraction peaks in Figure 2A as observed by X-ray diffraction (XRD) indicated the crystalline nature of the F-AuNPs. The value of each peak in the XRD patterns, as observed at 38.13°, 44.43°, 64.66°, and 77.66°, showed the reflection of a crystalline metallic gold particle with values of (111), (200), (220), and (311), respectively (Figure 2A). The above results concur with the XRD patterns of gold nanoparticles reported previously [30,38]. Finally, we also determined the presence of gold as a major constituent in the F-AuNPs by energy dispersive X-ray diffraction (EDX) (Figure 2B). Among the major peaks in the spectrum, the peak appearing at 2.2 keV is a characteristic peak of gold present in the F-AuNPs, whereas the peak at 8.2 keV is that of Cu available from the grid used. The elemental composition of F-AuNPs has also been analyzed previously using EDX with similar peak profiles [30].

### 3.2. Determination of Minimum Inhibitory Concentration (MIC) of F-AuNPs and Growth Properties of P. aeruginosa in the Presence of F-AuNPs

**Figure 3:**
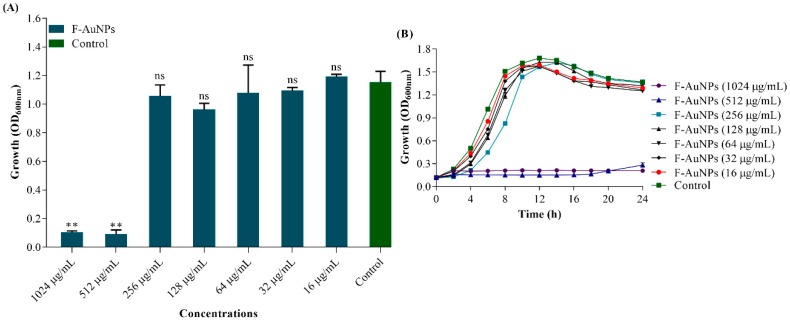
(**A**) Determination of minimum inhibitory concentration of F-AuNPs at 600 nm wavelength and (**B**) growth curve analysis of *P. aeruginosa* in the presence of different concentrations of F-AuNPs at every 2 h time interval the OD at 600 nm wavelength. The experiments were performed in triplicate with two independent cultures. ** *p <* 0.01 considered as significant and ns indicates non-significant as compared to the control (not treated by F-AuNPs).

Before investigating the start of biofilm inhibition and the virulence attenuating properties of synthesized F-AuNPs, the MIC was determined using different concentrations (ranging from 16–1024 µg/mL) of F-AuNPs. The MIC was determined by measuring the OD of bacterial cell growth at 600 nm after 24 h of incubation under shaking conditions (120 rpm). Figure 3A clearly shows a significant inhibition of *P. aeruginosa* growth at 512 and 1024 µg/mL of F-AuNPs. Hence, based on the above results, the MIC value of F-AuNPs for *P. aeruginosa* was assigned as 512 µg/mL (Figure 3A). The growth profile of *P. aeruginosa* in the presence of different concentrations (ranging from 16–1024 µg/mL) of F-AuNPs was also determined by measuring the OD_600_ at 2 h time intervals up to 24 h during incubation under agitation (120 rpm). The growth pattern of *P. aeruginosa* in the presence of each subinhibitory concentration (sub-MIC) of F-AuNPs was found to be similar to the control (Figure 3B). Thus, based on the above results, it is evident that F-AuNPs at sub-MIC levels caused a bactericidal effect to bacterial cells throughout the experiment.

### 3.3. Biofilm Inhibition Properties of F-AuNPs

**Figure 4:**
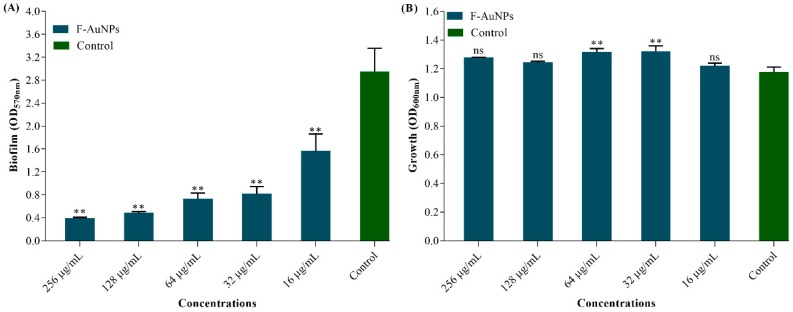
Biofilm inhibition properties of different concentration of F-AuNPs. (**A**) Biofilm assays and (**B**) growth analysis of *Pseudomonas aeruginosa*. The experiment was repeated three times for each concentration of F-AuNPs. ** *p <* 0.01 considered as significant and ns indicates non-significant as compared to the control (not treated by F-AuNPs).

The anti-biofilm activity of F-AuNPs against *P. aeruginosa* was determined by crystal violet staining assays and OD measurements at 570 nm. As shown in Figure 4A, the sub-MIC levels of F-AuNPs when incubated with *P. aeruginosa* cells cultured overnight (initial turbidity of 0.05 at 600 nm) exhibited concentration-dependent biofilm inhibition. In comparison to the non-treated control, F-AuNPs at 128 µg/mL and 256 µg/mL concentrations showed approximately 86% and 84% biofilm inhibition, respectively. The minimum biofilm inhibitory concentration (MBIC) of F-AuNPs for *P. aeruginosa* was therefore assigned as 128 µg/mL (Figure 4A). The growth property of *P. aeruginosa* in the presence of sub-MIC of F-AuNPs was also checked by measuring the OD at 600 nm (Figure 4B). The results showed that there were no bactericidal effects at each concentration of F-AuNPs when incubated under static conditions (without shaking).

**Figure 5:**
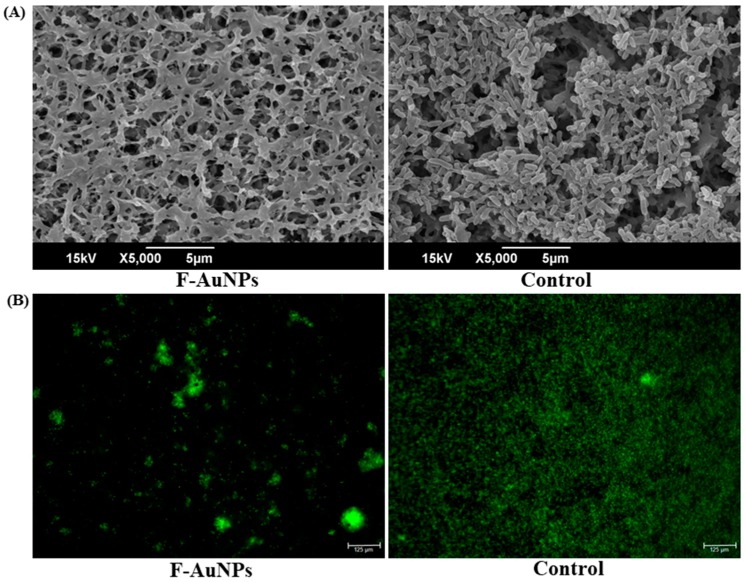
Microscopic examination of biofilm cells and biofilm architecture after 24 h of incubation with F-AuNPs (256 µg/mL). (**A**) SEM image and (**B**) fluorescence image of biofilm cells.

Furthermore, the effects of F-AuNPs on cell morphology as well as biofilm architecture were examined using a scanning electron microscope (SEM) and fluorescence microscopy for the 24 h treated and non-treated cells (Figure 5). The results of SEM analysis of the cell culture incubated along with F-AuNPs (256 µg/mL) for 24 h showed a lack of cells attached to the nylon surface, whereas the cell culture not treated with F-AuNPs showed dense layers of sessile cells adhered to the nylon surface (Figure 5A). The results obtained from fluorescence microscopy using acridine orange dye (10 µg/mL) showed a significant reduction of green fluorescence in the presence of F-AuNPs (256 µg/mL), while non-treated cells (control) exhibited intense green fluorescence (Figure 5B). Fluorescence microscopy analysis also confirmed that F-AuNPs inhibited the attachment of cells to the glass surface as compared to the control. Thus, based on crystal violet assays, SEM, and fluorescence microscopy studies, it can be concluded that F-AuNPs disrupted the attachment of sessile cells to surfaces, which initiated the formation of biofilms.

**Figure 6:**
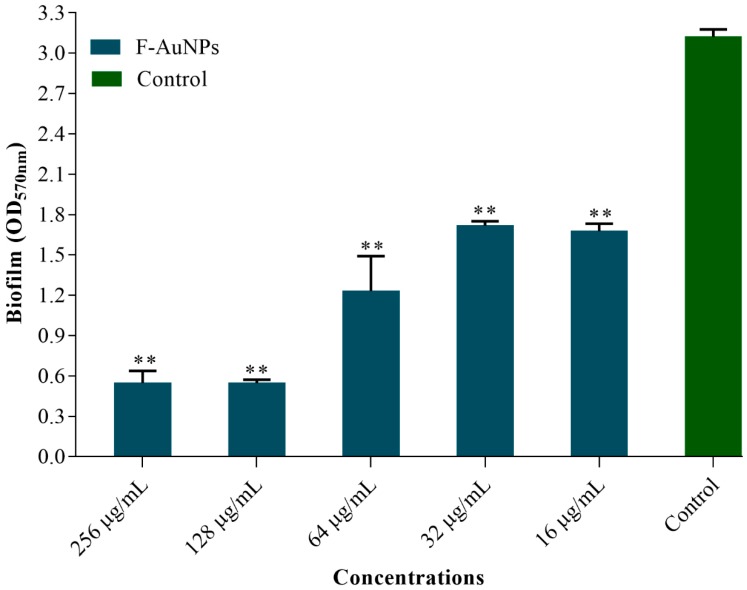
Dispersion of established mature biofilm of *P. aeruginosa* in the presence of F-AuNPs. The 24 h established matured biofilm was analyzed by crystal violet staining method and OD measurement at 570 nm. The experiment was repeated three times for each F-AuNP concentration. ** *p <* 0.01 versus the control (not treated by F-AuNPs).

Apart from the inhibition of biofilm formation at the initial stage by F-AuNPs, the dispersion of mature biofilm established by *P. aeruginosa* was also studied (Figure 6). The 24 h old established mature biofilm was treated with different concentrations (ranging from 16–256 µg/mL) of F-AuNPs. The results showed that higher concentration (from 128–256 µg/mL) exhibited stronger dispersion of established mature biofilm, as compared to the lower concentration (16–64 µg/mL). The minimum biofilm eradication concentration (MBEC) of F-AuNPs on pre-formed mature *P. aeruginosa* biofilm was therefore selected as 128 µg/mL.

### 3.4. Antivirulence, Antihemolytic and Protease Inhibitory Activity of F-AuNPs

**Figure 7:**
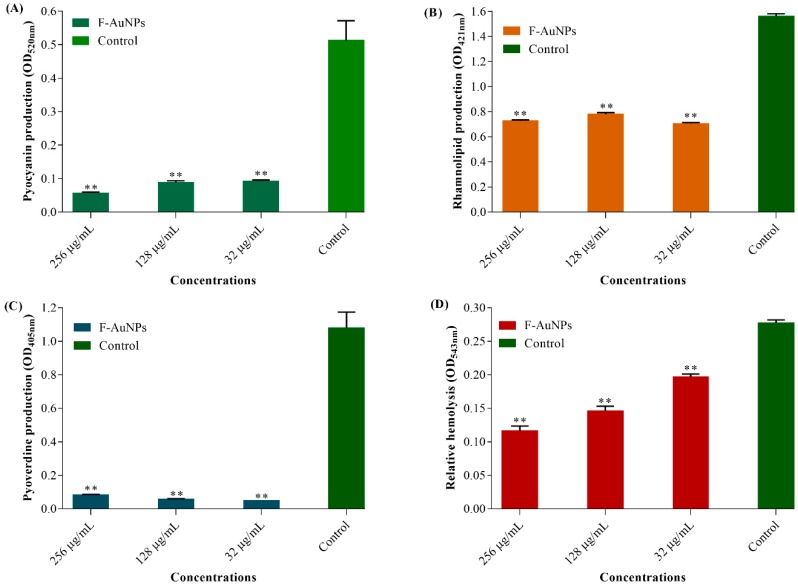
Effect of F-AuNPs on the production of virulence factors and hemolytic activity in *P. aeruginosa*. (**A**) Production of pyocyanin, (**B**) production of rhamnolipid, (**C**) production of pyoverdine, and (**D**) hemolytic activity. The determination of virulence factor production and hemolytic activity from the F-AuNPs treated sample were carried out as a relative value in comparison to the control. All the experiments were performed in triplicate. ** *p <* 0.01 versus the control (not treated by F-AuNPs).

The sub-MICs of F-AuNPs were also checked for inhibitory effects on the bacterial production of several virulence factors during biofilm formation that are essential for colonization and pathogenesis. Production of pyocyanin from *P. aeruginosa* in the presence of different concentrations of F-AuNPs was determined spectrophotometrically at 520 nm. The results showed a significant loss in the inhibition of pyocyanin, in which pyocyanin production at 32, 128, and 256 µg/mL concentrations of F-AuNPs were found to be approximately 79.4%, 81.9%, and 87.7%, respectively (Figure 7A). Similarly, the amount of rhamnolipid production was determined by using an orcinol colorimetric assay and OD measurements at 421 nm. Concentrations of 32, 128, and 256 µg/mL of F-AuNPs reduced rhamnolipid production by 54%, 50%, and 53%, respectively, which represents almost equal inhibition at all concentrations tested (Figure 7B). Production of another virulence factor, pyoverdine, which is one of the siderophores required for iron acquisition from the environment was also checked in the presence of different sub-MICs of F-AuNPs. Pyoverdine production was measured directly in the supernatant at a wavelength of 405 nm. The results showed that at 256 µg/mL, inhibition of pyoverdine production by *P. aeruginosa* was 91.6%, whereas, at 128 µg/mL and 32 µg/mL, bacterial pyoverdine generation was inhibited by almost 95% (Figure 7C).

**Figure 8:**
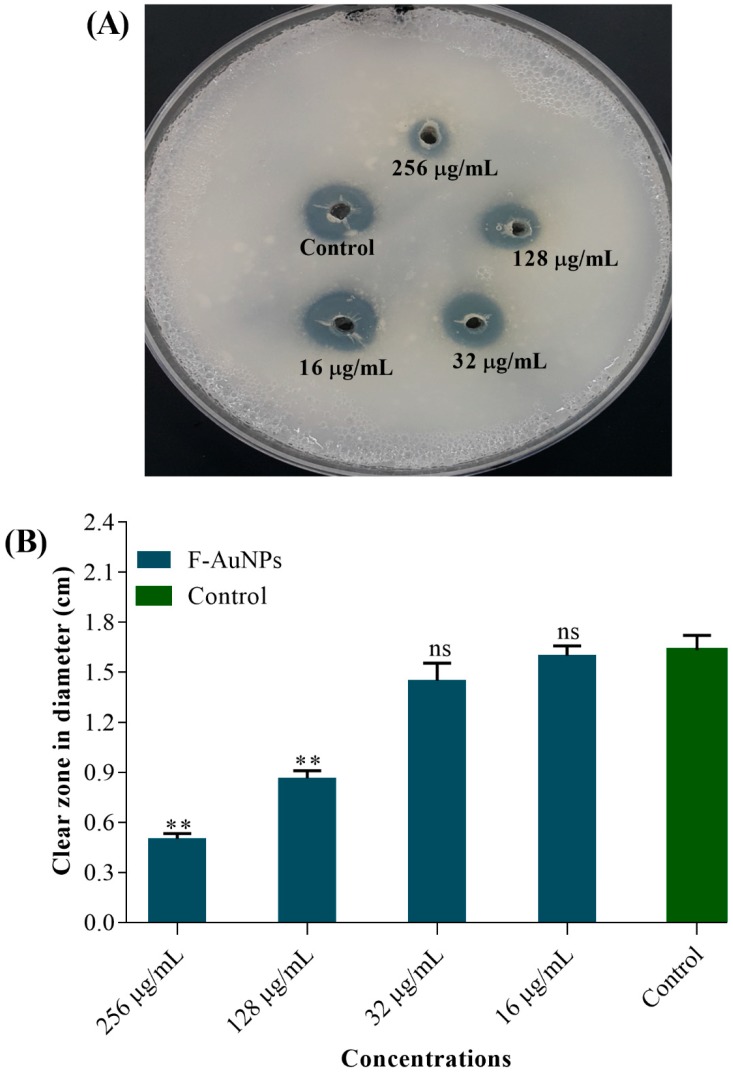
Protease inhibitory activity of F-AuNPs at sub-MICs in *P. aeruginosa*. (**A**) The image of the casein-containing agar plate showing protease activity, and (**B**) diameter (cm) of clear zones appearing around the holes. All the experiments were performed in triplicate. ** *p <* 0.01 considered as significant, ns indicates non-significant as compared to the control (not treated by F-AuNPs).

In addition to the virulence factor production assays, we checked the hemolytic activity of *P. aeruginosa* in the presence of different sub-MICs of F-AuNPs. Bacterial cell cultures treated with F-AuNPs were mixed with diluted RBCs, followed by 1 h of incubation at 35 °C. The hemolyzed RBCs present in the supernatant were monitored at 543 nm. The results showed that with F-AuNPs at concentrations of 32, 128, and 256 µg/mL, the inhibition of hemolytic activity was 29%, 47.5%, and 59%, respectively (Figure 7D). Previous reports identified the fact that synthesis and production of protease enzymes from the cells are also functionally important in the pathogenesis of *P. aeruginosa* [39,40]. Hence, the production of protease enzymes in the presence of sub-MICs of F-AuNPs on casein-containing agar plates was assayed, and the results were revealed by the diameter (cm) of clear zones appearing around the treatment-loaded agar holes. As shown in Figure 8A,B, the maximum inhibitory effect of F-AuNPs over the bacterial production of proteases was exhibited at high concentrations (128 and 256 µg/mL).

### 3.5. Motility Impairment Properties of F-AuNPs

**Figure 9:**
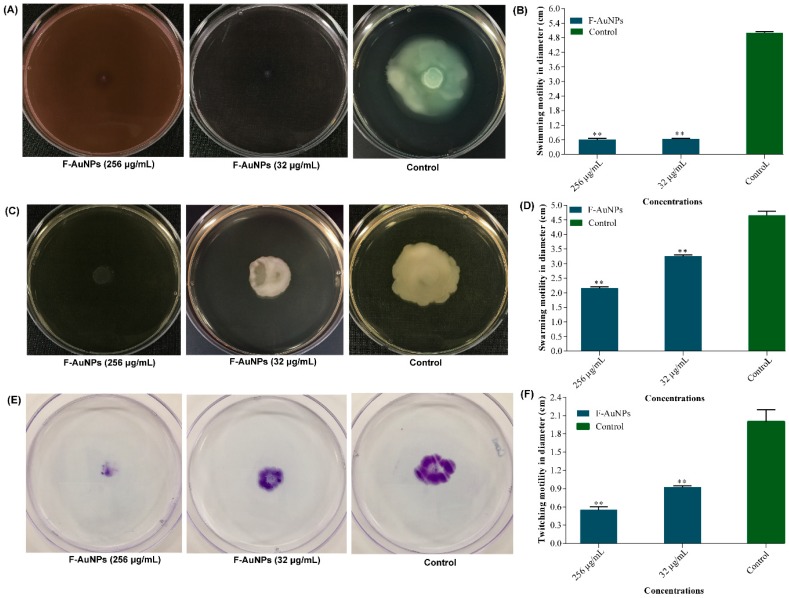
Motility inhibiting properties of F-AuNPs in *P. aeruginosa*. (**A**) Swimming motility image, (**B**) swimming motility values, (**C**) swarming motility image, (**D**) swarming motility values, (**E**) twitching motility image, and (**F**) twitching motility values. All the experiments were performed in triplicate. ** *p <* 0.01 versus the control (not treated by F-AuNPs).

Different types of motilities, such as swimming, swarming, and twitching, exhibited by *P. aeruginosa* have been well studied, and these motilities play a significant role in biofilm formation as well as infection of host cells [41,42,43]. The various types of motilities are due to the presence of surface appendages on *P. aeruginosa* such as flagellae and pili [42,43]. In the present study, the activity of F-AuNPs at sub-MIC levels (32 and 256 µg/mL) on various types of motilities of *P. aeruginosa* such as swimming, swarming, and twitching was studied on agar plates. Swimming motility was monitored in Bacto agar (0.3%) media containing NaCl (1%) and tryptone (0.25%). As shown in Figure 9A,B, flagellar-mediated swimming motility was completely inhibited in comparison to the control (absence of the drug).

Similarly, another type of flagellar motility known as swarming was investigated on the surface of Bacto agar (0.4%) plates in LB broth supplemented with glucose (0.5%) and casamino acids (0.5%). As shown in Figure 9C,D, swarming motility was also inhibited in a concentration-dependent manner, with values of approximately 30% and 53% at concentrations of 32 and 256 µg/mL, respectively. Furthermore, the present study also monitored type IV pili-mediated twitching motility using solid Bacto agar (1.5%) prepared in LB broth containing glucose (30 mM) and casamino acids (0.2%). In contrast to swarming and swimming, the twitching assay was monitored by staining with crystal violet (0.1%). The results showed that twitching motility was found to be significantly inhibited in a concentration-dependent manner (Figure 9E,F). The results revealed twitching motility inhibition of almost 72% at 256 µg/mL, and almost 54% at 32 µg/mL concentration of F-AuNPs (Figure 9F). Collectively, the present results indicated that F-AuNPs effectively controlled the different motility modes of *P. aeruginosa*.

## 4. Discussion

Several strategies have been developed in order to combat antibiotic resistance and related infections caused by pathogenic bacteria [44,45,46,47]. Besides targeting resistance enzyme synthesis and efflux pump function, these strategies also aim for inhibition of biofilm formation and attenuation of virulence factors produced by pathogenic bacteria, hence reducing selection pressure and preventing future risk of resistance [6,48,49]. With the recent development of nanotechnology, noble metal-based nanoparticles such as AuNPs in a size range of 1–100 nm, with easy surface modifications, high compatibility, and low toxicity have been recognized as a promising antibiofilm agent, as well as an effective drug delivery system [50,51,52,53,54]. Modern synthesis techniques of AuNPs have shifted from physical and chemical methods to biological approaches, which are mediated by plants, algae, and microorganisms for improvements in modification, stability, economic benefit, production scale-up, and environmental friendliness [55,56,57]. In fact, it is those biocompatible, biodegradable, and non-toxic active compounds such as polysaccharides, proteins, and phenolics enriched in these biomaterials that initiate both the bio-reduction of metallic ions to NPs and their stabilization [58,59,60]. Specifically, in AuNPs, biopolymer-based biosynthesis has even been found to be more efficient than other methods [61]. In the present study, fucoidan, which is a sulfonated polysaccharide sourced from various brown seaweed species with significant bioactivities, including antimicrobial, antioxidant, anti-inflammatory, and anti-cancer roles, was used to synthesize stabilized-AuNPs [25,26]. Owing to the availability and relatively high purity of fucoidan (≥95%), the use of commercial fucoidan products, which are extracted from *Fucus vesiculosus,* is recommended as an economically beneficial approach in nanoparticle biosynthesis [62,63]. Several crucial characterization analyses involving UV-vis spectrophotometry, FTIR, DLS, FE-TEM, EDX, and XRD were carried out involving the synthesized F-AuNPs, and the results are presented in Figure 1 and Figure 2. The prepared F-AuNPs were spherical in shape and approximately 15 to 119 nm in size (with an average size of ~53 nm), with high stability and high water solubility, and can be used for subsequent experiments involving anti-biofilm functions.

The resultant F-AuNPs were examined for functional potential in inhibiting biofilm formation and virulence factor production by *P. aeruginosa*. The MIC value was first determined to be 512 µg/mL. High concentrations (i.e., MIC and > MIC) of F-AuNPs exhibited bactericidal activity, while lower concentrations (i.e., sub-MICs) were effective in preventing biofilm establishment, virulence factor production, and eradicating pre-existing mature biofilm. The antibacterial effect of high concentrations of F-AuNPs was also found in several other biogenic NPs derived from either fucoidan or Au. For example, fucoidan was previously used to prepare silver NPs (AgNPs), and results showed that F-AgNPs exhibited significant antibacterial activity against *Klebsiella pneumoniae* [27]. Meanwhile, AuNPs synthesized from *Lignosus rhinocerotis* sclerotial extract and chitosan also induced growth inhibition of a wide range of foodborne bacteria such as *Bacillus* sp., *Escherichia coli*, *P. aeruginosa*, and *Staphylococcus aureus* [64]. Studies have also found that several Gram-negative bacterial species are more susceptible to antibacterial agents than their Gram-positive counterparts due to the lack of a thick peptidoglycan wall, which allows higher uptake of these agents [65,66]. Collectively, the bactericidal effects of F-AuNPs at high concentration have added to the potential use of F-AuNPs as an effective antibacterial agent against *P. aeruginosa*.

The inhibition of formation and eradication of biofilm, as well as the production of other virulence factors by biosynthesized F-AuNPs, were mainly identified at sub-MIC levels. In attempts to lower the selection pressure for resistance, targeting biofilm formation and genetic expression of other important virulence factors are considered to be approaches with the most potential, and which are commonly involved in the application of nanotechnology. NPs of nano-scale sizes and high stability are capable of inhibiting biofilm formation and damaging pre-existing mature biofilm structures mostly formed on infected living tissues and nosocomial systems [12]. In the present study, F-AuNPs exhibited antibacterial activity at a concentration of 512 µg/mL, while exhibiting antibiofilm activity and biofilm eradication activity at 128 µg/mL. Microscopic observations by SEM and fluorescence microscopy also confirmed the effectiveness of F-AuNP treatment, in which the presence of F-AuNPs significantly disrupted 24 h old biofilm thickness and architecture, in comparison with the control without F-AuNPs. Similar results were obtained when AuNPs prepared from baicalein and from apple extract were applied to *P. aeruginosa* biofilms [54,67]. Moreover, crystal violet assays and microscopic observations clearly confirmed the inhibitory and eradicating efficacy of F-AuNPs at sub-MIC levels against *P. aeruginosa* biofilm.

Along with biofilm formation, *P. aeruginosa* is known to produce a wide array of virulence factors actively engaged in chronic infections [68]. Of all of these factors, rhamnolipid, pyocyanin, pyoverdine, hemolysins, protease, and cell motilities were selected to examine their production under sub-MIC levels of F-AuNPs. Results showed that production of pyoverdine, pyocyanin, and rhamnolipid were significantly reduced in the presence of F-AuNPs at sub-MIC levels. With equal amounts of F-AuNP, hemolytic activity was reduced in a concentration-dependent manner. Green-blue pigmented pyocyanin essentially causes oxidative stress and cytotoxicity to the host tissues; pyoverdine maintains the iron requirement for bacterial survival and growth; rhamnolipid is essential for motility and biofilm formation; hemolysins cause rupture of host RBCs; and proteases damage host immune systems. Therefore, reduction of these crucial virulence factors can be considered to effectively attenuate the pathogenesis and colonization of *P. aeruginosa* without affecting bacterial growth or initiating resistance selection [69,70,71,72,73].

To the best of our knowledge, the inhibitory effects of F-AuNPs towards *P. aeruginosa* virulence factors at both the phenotypic and genetic levels have remained unknown. So far, only AuNPs synthesized from ectomycorrhizal fungi were found to completely inhibit pyocyanin production by *P. aeruginosa* [74]. Therefore, the finding of anti-virulence activity of F-AuNPs against bacteria, as obtained in the present study, has provided essential insights for the future application of F-AuNPs in controlling *P. aeruginosa* pathogenesis, as well as against biofilm-related infections.

Motility and attachment of bacterial planktonic cells to biotic or abiotic surfaces are known to set the primary platform for subsequent stages of biofilm formation. Therefore, this transition phase is also considered to be a common target in preventing biofilm formation [75]. In *P. aeruginosa*, swimming, swarming, and twitching motilities are largely mediated by pili IV and flagellae. Here, in the present study, compared to the control, sub-MIC levels of F-AuNPs were able to impair all types of motilities, with the most significant inhibitory effect being observed in swimming and twitching. Likewise, sub-MICs of AuNPs prepared from cinnamon oil, betulinic acid, baicalein, and curcumin have also been reported to target the motility of planktonic *P. aeruginosa* cells, causing a notable reduction in biofilm biomass up to 89% [10,60,75,76].

## 5. Conclusions and Future Perspectives

Biofilm formation emerged in numerous bacteria as a drug resistance mechanism, and has remained a great threat to the global population to date. Among current novel treatments, noble NPs, such as AuNPs, have been recognized for their significant anti-biofilm efficacy. However, studies on the efficacy of AuNPs synthesized from biological sources have been limited. For this reason, the present study employed fucoidan, a sulfonated polymer sourced from marine seaweed, as a stabilizing and reducing agent to synthesize AuNPs. As the biosynthesized F-AuNPs were characterized as stable and water-soluble, they were further evaluated for anti-biofilm potential against *P. aeruginosa*. F-AuNPs at high concentration killed the bacterial cells, whereas F-AuNPs at sub-MIC inhibited biofilm formation and eradicated mature, established, 24 h old biofilm. The sub-MICs of F-AuNPs also suppressed the production of several virulence factors by *P. aeruginosa*. Inhibition of *P. aeruginosa* hemolytic activity by F-AuNPs was in a concentration-dependent manner. Furthermore, additional activities of the F-AuNPs extended towards different motility properties of *P. aeruginosa*. The results showed that F-AuNPs impaired the swarming, swimming, and twitching motilities at the sub-MIC level. Thus, it can be concluded that the present biosynthesized F-AuNPs constitute a stable, water-soluble anti-biofilm and anti-virulence drug against *P. aeruginosa*. In the long term, future studies are required for more in-depth understanding regarding F-AuNPs’ inhibitory mechanisms towards bacterial biofilm, virulence factors, and motility at the molecular level. The antibacterial activity of F-AuNPs should also be researched for its mode of action, because the negatively-charged F-AuNPs might exhibit bactericidal effects differently in comparison with positively-charged NPs such as chitosan NPs. In addition, biocompatibility and efficacy of F-AuNPs should be examined in animal models such as *Caenorhabditis elegans* for potential clinical use. Furthermore, as *P. aeruginosa* biofilm formation is associated with a wide variety of nosocomial infections, the application of F-AuNP treatment in biomedical settings could be a promising solution. Consequently, further investigation regarding to F-AuNP efficacy and multi-species biofilm formation is required.
